# Regularized regression outperforms trees for predicting cognitive function in the Health and Retirement Study

**DOI:** 10.1016/j.mlwa.2025.100694

**Published:** 2025-07-12

**Authors:** Kyle Masato Ishikawa, Deborah Taira, Joseph Keaweʻaimoku Kaholokula, Matthew Uechi, James Davis, Eunjung Lim

**Affiliations:** a Department of Quantitative Health Sciences, John A. Burns School of Medicine, University of Hawaii at Manoa, 651 Ilalo St, Honolulu, HI, USA; b Department of Pharmacy Practice, The Daniel K. Inouye College of Pharmacy, University of Hawaii at Hilo, 677 Ala Moana Blvd, Honolulu, HI, USA; c Department of Native Hawaiian Health, John A. Burns School of Medicine, University of Hawaii at Manoa, 677 Ala Moana Blvd, Honolulu, HI, USA; d Department of Geriatric Medicine, John A. Burns School of Medicine, University of Hawaii at Manoa, 347 N Kuakini St, Honolulu, HI, USA

**Keywords:** Machine learning, Cognitive function, Linear regression, Tree-based modeling

## Abstract

**Background:**

Generalized linear models have been favored in healthcare research due to their interpretability. In contrast, tree-based models, such as random forest or boosted trees, are often preferred in machine learning (ML) and commercial settings due to their strong predictive performance. However, for clinical applications, model interpretability remains essential for actionable results and patient understanding. This study used ML to detect cognitive decline for the purpose of timely screening and uncovering associations with psychosocial determinants. All models were interpreted to enhance transparency and understanding of their predictions.

**Methods:**

Data from the 2018 to 2020 Health and Retirement Study was used to create three linear regression models and three tree-based models. Ten percent of the sample was withheld for estimating performance, and model tuning used five-fold cross validation with two repeats. Survey frequency weights were applied during tuning, training, and final evaluation. Model performance was evaluated using RMSE and R^2^ and interpretability was assessed via coefficients, variable importance, and decision trees.

**Results:**

The elastic net model had the best performance (RMSE = 3.520, R^2^ = 0.435), followed by standard linear regression, boosted trees, random forest, multivariate adaptive regression splines, and lastly, decision trees. Across all models, baseline cognitive function and frequency of computer use were the most influential predictors.

**Conclusion:**

Elastic net regression outperformed tree-based models, suggesting that cognitive outcomes may be best modeled with additive linear relationships. Its ability to remove correlated and weak predictors contributed to its balance of interpretability and predictive performance for this particular dataset.

## Introduction

Parametric regression models are a standard in healthcare research due to their interpretability, but external validation (real-world performance) and generalizability are essential to provoke change in practice (Rose, 2020). For example, the significant odds ratio of a logistic regression model may be interpreted with less importance if the same model has a sensitivity of 0.6 on real world data. For applications such as disease diagnosis or screening, performance would be an additional requirement to explainability. To solve this problem, machine learning (ML) utilizes a technique called *validation* to determines a model’s performance on unseen, or *real-world*, data. ML models are becoming more prevalent in healthcare for tasks such as image recognition or natural language processing due to their performance and ability to process structured and unstructured data (Jiang et al., 2017).

Mild cognitive impairment (MCI) is the transitional period between normal cognitive aging and more serious forms of dementia. Early detection of cognitive decline enables timely lifestyle interventions that may help preserve cognitive function. It also allows families to make informed decisions and plan effectively to support the individual’s future needs (He et al., 2023). Every year for the past decade, publications which apply predictive ML to the domain of MCI have been on the rise (Liu et al., 2025). In a systematic review by Grueso and Viejo-Sobera (2021), support vector machines (SVM) and convolutional neural networks were the most common models applied to magnetic resonance images (MRI) and positron emission tomography to detect cognitive decline ranging from MCI to Alzheimer’s disease (AD). These models achieved an average accuracy of 75.4 % and 78.5 %, respectively. The authors noted that combining complex models with multidomain features such clinical, cognitive, genetic, and behavioral aspects, achieved the best performance. Within ML workflows that use complex imaging data, intermediate steps need to be taken to get the data into tabular format such as calculating surface area, detecting features, or counting objects (Grueso & Viejo-Sobera, 2021).

ML models can be grouped into two categories – *white-box* models that are explainable and *black-box* models whose inner mechanisms cannot be generalized into any equation or form (Linardatos et al., 2020). The latter requires post hoc methods such as *variable importance* to be calculated in order for the model to be interpreted (Sierra-Botero et al., 2024). Although the model’s inner workings are still unknown, each predictor’s importance can be interpreted with respect to the model’s performance, thereby turning the black-box model into a *grey-box* model. Research regarding “explainable machine learning” has been on the rise in the past decade (Linardatos et al., 2020) and shows that explainability is essential for performant models to be adopted into healthcare. In their 2025 study, Vlontzou et al. (2025) created an ML framework for diagnosing MCI and AD. Quantitative measurements such as hippocampal volume and cortical thickness were extracted from MRIs and placed in tabular format before being inputted into SVM and ensemble models. Variable importance calculations bring transparency to model predictions because their inputs mirror the same features clinicians use in their decision making.

Decision trees are the foundation for more complex models such as random forest or boosted trees. The model starts with a root *node*, or decision, that splits the outcome data by a predictor that explains the most variation in the outcome. Each subset of data *branches* into another node to be split again. This process continues until the node perfectly separates the outcome class or the number of observations at the node drops below a specified minimum. Algorithms such as C5.0 prevent overfitting by using cross-validation and pruning to eliminate branches that do not perform well (Pandya & Pandya, 2015). The result is a decision tree that explains most of the outcome variation without overfitting to the data. Although a decision tree provides paths that are easily interpretable, the model itself is considered a *weak learner* because it does not inference or predict well.

A random forest is a collection, or *ensemble*, of many decision trees. Each tree is built by selecting a random subset of predictors at each node to prevent overfitting. Therefore, each node chooses the predictor and cutoff value that explains the most variation in the outcome variable. If a node is downstream of another, the split depends on how the previous node splits the data and which subset of predictors is available to the current node. This randomness of predictors has a regularization effect, meaning that the model does not overfit to strong predictors at each node. All trees are independent of one another and considered *weak learners*. Random forest can be coupled with variable importance analysis to provide clear insights into the factors driving predictions. For example, Ooka et al. (2021) used this approach to identify key predictors of hemoglobin A1c changes in large-scale health check-up data, while Loef et al. (2022) applied it to uncover long-term health predictors in a 30-year cohort study, helping to clarify which variables most influenced their outcomes. A boosted tree model creates trees similarly to the random forest model, but with an important difference – each tree is fitted to the residuals of all previously created trees. This means that if previous trees performed poorly in certain areas, the next tree will try to correct those shortcomings. In ML competitions hosted on Kaggle, ensemble methods such as random forest outperform traditional regression models, and boosted methods such as boosted trees perform even better than random forest (Bojer & Meldgaard, 2021). In 2015, boosted trees were the most commonly used model, featured in 17 of the 29 winning Kaggle solutions (Chen & Guestrin, 2016). Accordingly, this study focuses on decision trees, random forests, and boosted trees – models that build upon one another, increasing in complexity and predictive performance.

This study aimed to evaluate the performance and interpretability of three regression-based models and three tree-based models in predicting follow-up cognitive function scores. The regression models include linear regression, elastic net, and multivariate adaptive regression splines (MARS), and the tree-based models include decision tree, random forest and boosted trees. It also provides plausible explanations for why models perform well relative to other models. For this comparison, we used data from the Health and Retirement Study (HRS) to predict change in cognitive function.

## Methods

### Study data

HRS is a longitudinal study documenting how middle-aged Americans transition to- and live out their retirement. The study’s design is multidisciplinary, incorporating elements of economics, sociology, psychology, epidemiology, and medicine. This study used data from the leave behind questionnaire of 2018 as baseline characteristics to predict cognitive function in 2020.

### Variables

The outcome variable was the 27-point Langa-Weir Classification Scale, which measured cognitive function with three main components – immediate and delayed recall of 10 words, counting backwards in increments of seven from 100, and counting backwards in increments of one from 20. Predictor variables included demographics and psychosocial factors such as social network support, social participation, and depression. These variables can be seen in more detail in Table 1, as well as their projected or weighted estimates for the entire older adult US population. Predictors were selected based on a literature review and what clinicians thought to be relevant to cognitive function. Even though some predictors had natural collinearity, such as activities of daily or instrumental living, these predictors were not removed during preprocessing, but rather, relied on the regularization ability of each model.

### Data analysis

Within each stratum provided by the HRS, 90 % of the data was randomly sampled and assigned to the *training* set, while the remaining 10 % was assigned to the *test* set and used solely for evaluating model performance. Within the training set, tuning was performed using five-cross fold validation with two repeats, resulting in ten estimates of performance for each set of model hyperparameters. The range of candidate hyperparameters were those suggested by the *dials* package (Kuhn & Frick, 2022). For each model, a set of 20 or 30 equally spaced hyperparameters were created withing the hyperparameter space. A list of candidate hyperparameters for tunable models are presented in Table 1, along with their finalized values. The hyperparameters finalized in the model were those that produced the lowest weighted root mean squared error (RMSE) during cross-validation. Since tuning can be computationally intensive, an ANOVA-based *racing* method was used to eliminate poorly performing hyperparameters at each iteration of tuning. Lastly, weighted metrics such as RMSE, mean absolute error (MAE), and R-squared value were calculated for each model’s predictions on the test set. Predictions from each model were plotted against their true observed values, with performance metrics overlaid on each plot.

Before modeling, the data had to be transformed to improve model performance and satisfy each model’s requirements. First, categorical variables with a substantial amount of missing data such as *children live within ten miles, financial transfer to kids,* and *financial transfer from kids* had an explicit “unknown” category assigned to their missing values. For all other missing values, a bagged trees imputation method was used to ensure complete data for regression models that cannot accommodate missing values. The only models that required further transformation were the elastic net and boosted trees models. Due to the regularization ability of the elastic net, numeric variables had to be normalized, centered, and scaled to a mean of zero and a standard deviation of one. Next, both the elastic net and boosted trees model required categorical variables to be in a numeric input matrix, so all categorical variables were transformed into dummy variables represented by ones and zeros. Although tree-based models can handle missing values, imputation was applied to their data for consistency. This was the minimal amount of preprocessing for each model suggested by Kuhn and Silge (2022) with the exception of removing correlated variables and variable with zero variance, which were not present. In the section “A Recommended Preprocessing,” Kuhn and Silge (2022) had also suggested normalizing the continuous predictors in the regression models for *potential* performance gains, but this was not implemented because interpretability was prioritized over marginal performance gains.

Variable importance for the random forest and boosted trees models was determined using the *permutation* method after each model was trained. Variable importance was assessed using the permutation method where the decrease in performance after shuffling each predictor’s values indicated its relative importance. This method of determining variable importance is considered *model agnostic* because it calculates importance *after* the model has been trained, making it applicable to any model.

**Fig. 1.**
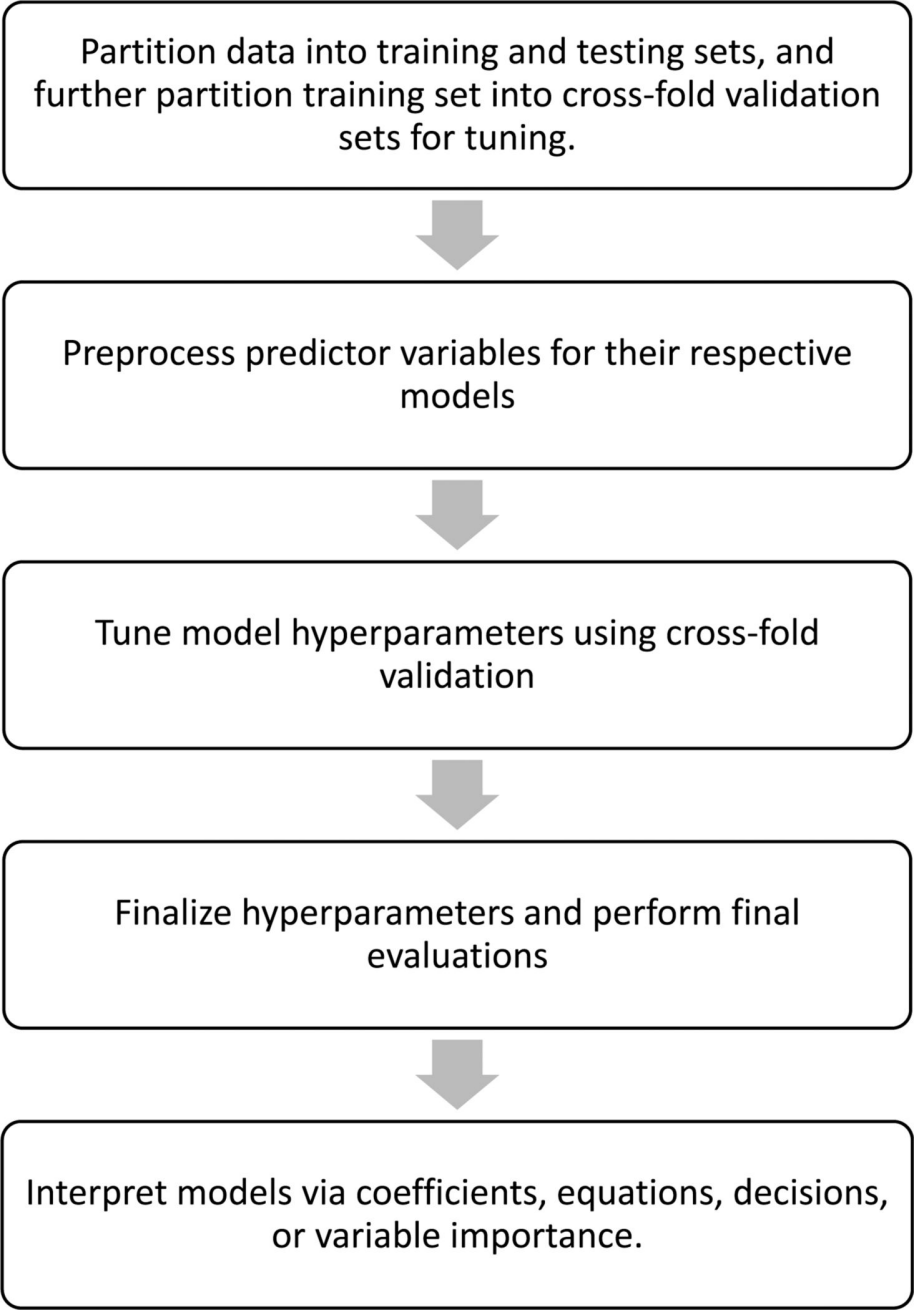
Overview of machine learning analysis.

All data analyses were performed in R version 4.4.1 using packages from the *tidyverse* (Wickham et al., 2019) and *Tidymodels* (Kuhn & Wickham, 2020). Code reproducibility was managed by the *targets* package (Landau, 2021). Weighted descriptive statistics were created using the *srvyr* package (Ellis & Schneider, 2019) and tables were produced using the *gtsummary* package (Sjoberg et al., 2021). An overview of the entire ML workflow previously described can be seen in Fig. 1. All computations were implemented on Apple’s M2 Max chip which had a 12-core central processing unit and 64 gigabytes of random-access memory.

## Results

**Fig. 2.**
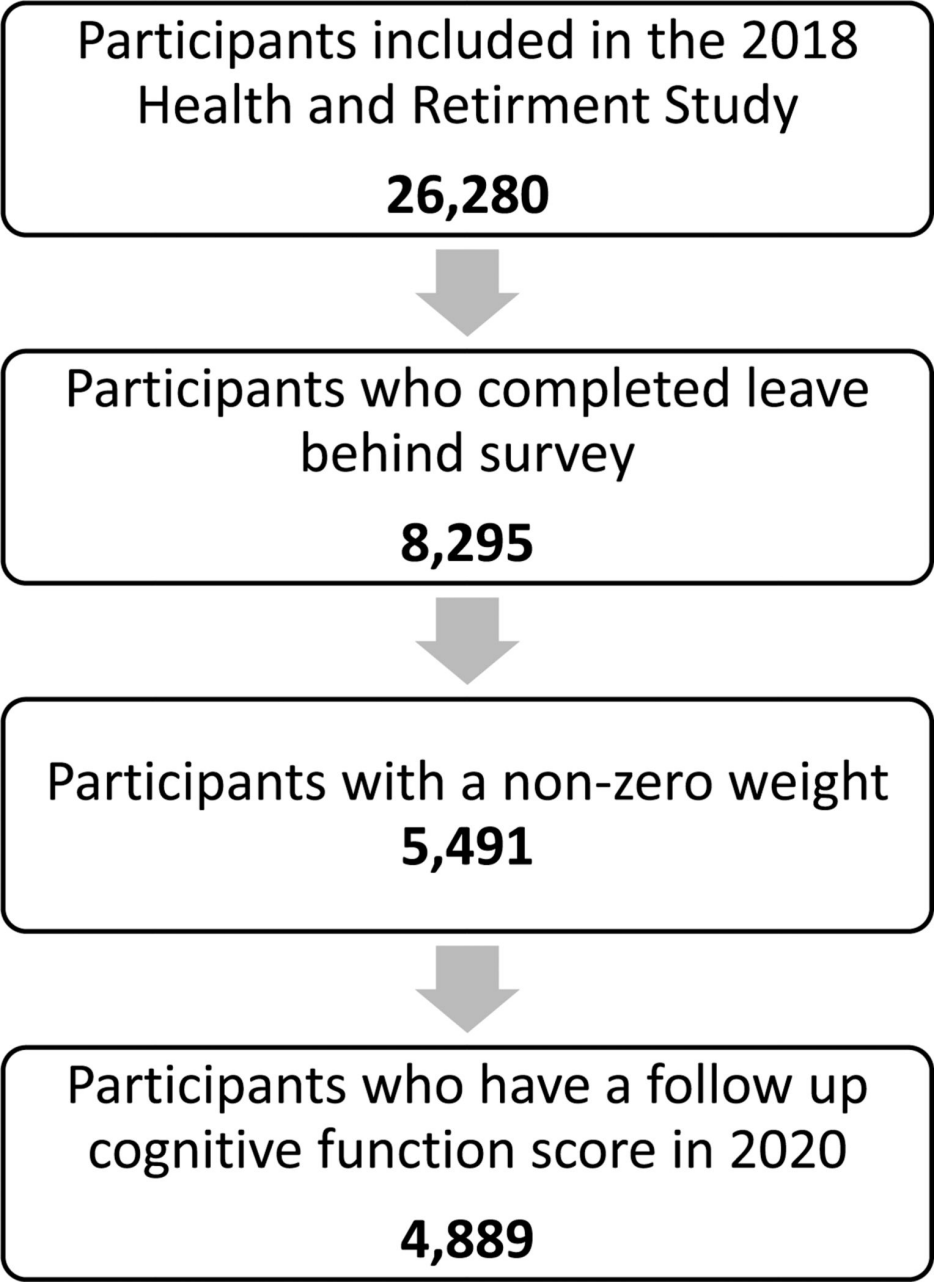
Study data flow chart.

The study data contained 4889 older participants who completed the 2018 leave-behind survey and had a 2020 cognitive function score (Fig. 2). The weighted and unweighted characteristics of the study population are presented in Table 1. The distributions of baseline- and follow-up cognitive function scores are shown in Figure S1. Both distributions are approximately normal, slightly skewed to the left, with modes of 17.

Further subgroup analysis was performed to see if there were differences in baseline cognition stratified by various demographic characteristics. Table 2 shows that individuals who are younger, female, White, non-Hispanic, married, above poverty, or have higher educational attainment tend to have higher baseline cognitive function scores. If the assumption can be made that follow-up cognitive function scores are correlated to baseline scores, then these demographic features will be good predictors for the ML models.

Tuning took up the largest amount of time in the ML workflow. The elastic net, RF, and boosted trees model took 4.2, 6.1, and 13.1 min to tune, respectively. Their range of candidate hyperparameters and their final values are listed in Table 3. Random forest benefited from having its trees trained in parallel, but boosted trees had to have its trees trained sequentially due to the fact that each subsequent tree was trained on the residuals of the previous tree. The final tree depth of boosted trees ended up being one, meaning that each tree was a *stump* and the minimum number of predictors was negligible. The linear regression and MARS models did not require tuning because the earth package (Milborrow et al., 2017) selected the number of predictors during training. However, the earth package notes that, “in the current implementation, building models with weights can be slow.” Hence, a single MARS model took 10.5 min to train. If there was an optimized algorithm for training MARS models with weights, then the MARS model would have been tuned for the degree of interactions. Tuning without weights determined that one degree of interaction was the optimal hyperparameter.

**Fig. 3.**
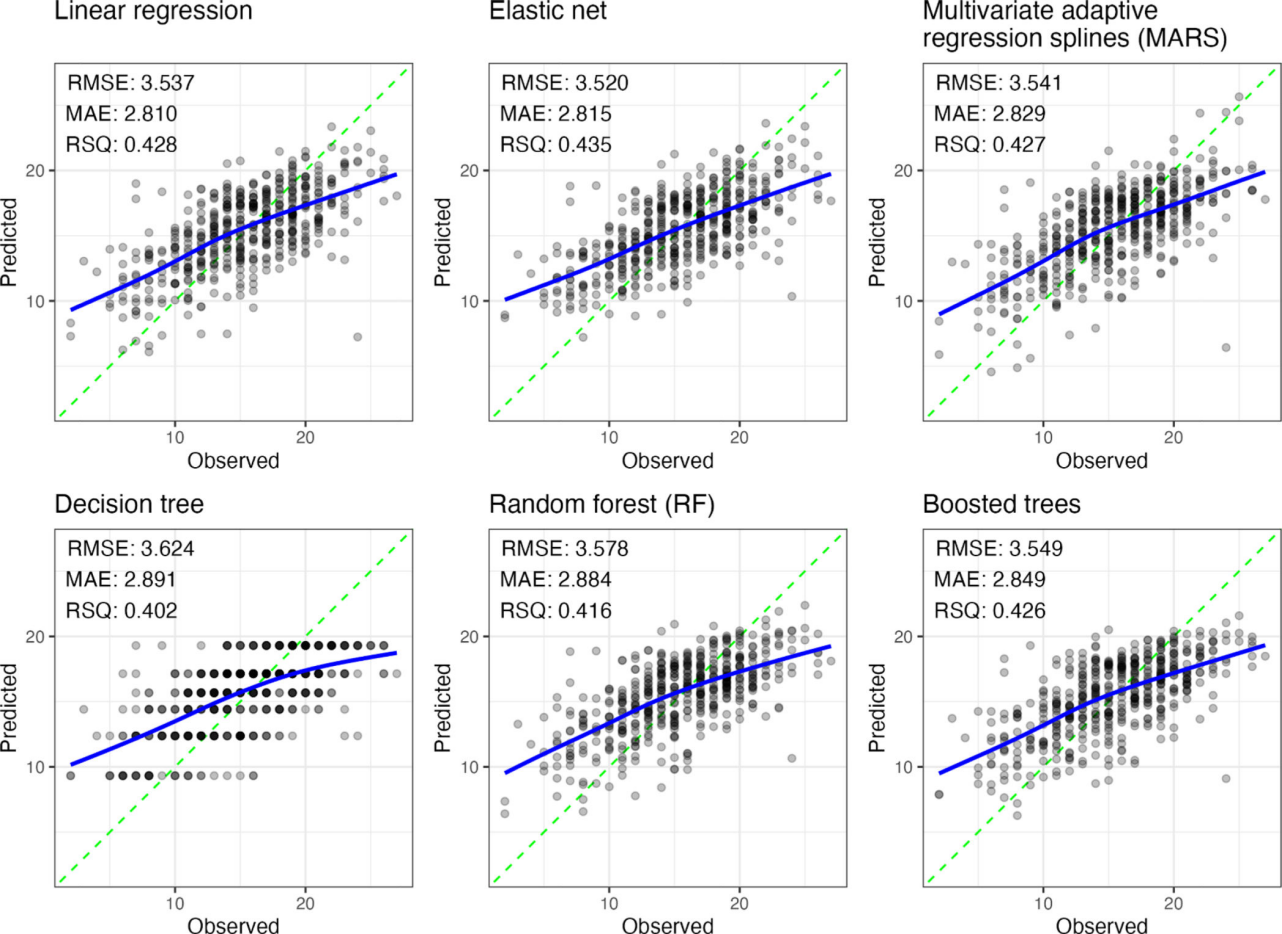
Performance of machine learning predictions on test set. Each plot shows the predicted versus the observed values from the test set, as well as their performance in the upper-left region.

The performance of each model is plotted in Fig. 3. Elastic net was the best performing model followed by linear regression, boosted trees, random forest, MARS, and lastly, decision trees. The predictions of the best performing model could be interpreted as, “the average error for all predictions is 2.8 cognitive function points” based on the MAE metric. The metrics of all final models were recorded from the cross-fold validation sets so that all measures of performance were comparable. Analysis of variance of RMSE and R^2^ values showed that there were differences in performance, and post hoc analysis determined that those differences lied between decision trees and all other models that performed better. Based on this result, the difference in performance of all models besides decision trees can be considered marginal.

The coefficient values for the linear regression and elastic net models are presented in Table 4. The variables selected from the elastic net model were inputted into a linear regression model so that coefficients could be estimated with raw values instead of centered and scaled values – this improved coefficient interpretability. Variable inflation factors (VIF) were calculated after modeling and confirmed variables with high correlation (VIF > 10). These 14 variables included the following groups - (1) Financial transfer to and from children; (2) number of unpaid helpers and family members; and (3) days and hours that participant got help from paid helpers, unpaid helpers, family, and non-family. Of the variables mentioned, the elastic net removed 10 of them and retained financial transfer to kids, and days or hours that participant got help from non-family or paid helpers. The model further removed predictors that were highly correlated or did not improve model performance. For example, there were four variables for positive or negative support from family or friends, of which only positive friend support was retained.

The MARS model did not perform as well the linear regression or elastic net models, but was able to present an interpretable set of rules for follow up cognitive function, shown in Equation 1. The equalities shown in the parentheses are hinge functions which can only be positive, indicated by the “+” sign. For example, for each year that a participant is younger than 69, their follow-up cognitive function decreases by 0.03, but for each year that a participant is older than 69, their follow-up cognitive function decreases by 0.12. In the end, the model retained 12 predictors. Characteristics that decrease follow-up cognitive function are a baseline cognitive function lower than 16, older age, being male or Black/African American, using Medicaid, attending non-religious activities, utilizing unpaid helpers, and negative friend support.

**Equation 1**. Equation of the multivariate adaptive regression splines model.


$$y_{2020\;\text{cognitive}\;\text{score}\;\text{function}\;}=13.73\;\text{(Intercept)}\;\;\;\;-0.60\times {\left(16-x_{2018}\;\text{cognitive}\;\text{score}\;\text{function}\;\right)}_{+}\;\;\;+0.42\times {\left(x_{2018}\;\text{cognitive}\;\text{score}\;\text{function}\;-16\right)}_{+}\;\;\;+1.20\times {\left(x_{2018}\;\text{cognitive}\;\text{score}\;\text{function}\;-24\right)}_{+}\;\;\;-0.030\times {\left(69-x_{\text{Age}}\right)}_{+}\;\;\;-0.13\times {\left(x_{\text{Age}\;}-69\right)}_{+}\;\;\;-0.55\times x_{\text{Male}\;}\;\;\;-0.52\times x_{\text{Medicaid}\;}\;\;\;-0.52\times x_{\text{Black/African}\;\text{American}\;}\;\;\;+0.44\times {\left(x_{\text{Frequency}\;\text{of}\;\text{computer}\;\text{use}\;}-5\right)}_{+}\;\;\;+0.22\times {\left(4-x_{\text{CES}-\text{D}}\right)}_{+}\;\;\;-0.14\times {\left(5-x_{\text{Frequency}\;\text{of}\;\text{non-religious}\;\text{activities}\;}\right)}_{+}\;\;\;+0.062\times {\left(24.59-x_{\text{Hours}\;\text{non-family}\;\text{helped}\;\text{last}\;\text{month}\;}\right)}_{+}\;\;\;+0.006\times {\left(x_{\text{Hours}\;\text{non-family}\;\text{helped}\;\text{last}\;\text{month}\;}-24.59\right)}_{+}\;\;\;-0.005\times {\left(x_{\text{Hours}\;\text{unpaid}\;\text{helpers}\;\text{helped}\;\text{last}\;\text{month}\;}-1\right)}_{+}\;\;\;+0.35\times {\left(x_{\text{Positive}\;\text{friend}\;\text{support}}\;-11\right)}_{+}\;\;\;-0.41\times {\left(x_{\text{Negative}\;\text{friend}\;\text{support}}\;-9\right)}_{+}\;\;\;+0.19\times {\left(7-x_{\text{Number}\;\text{of}\;\text{chronic}\;\text{conditions}}\right)}_{+}$$

**Fig. 4.**
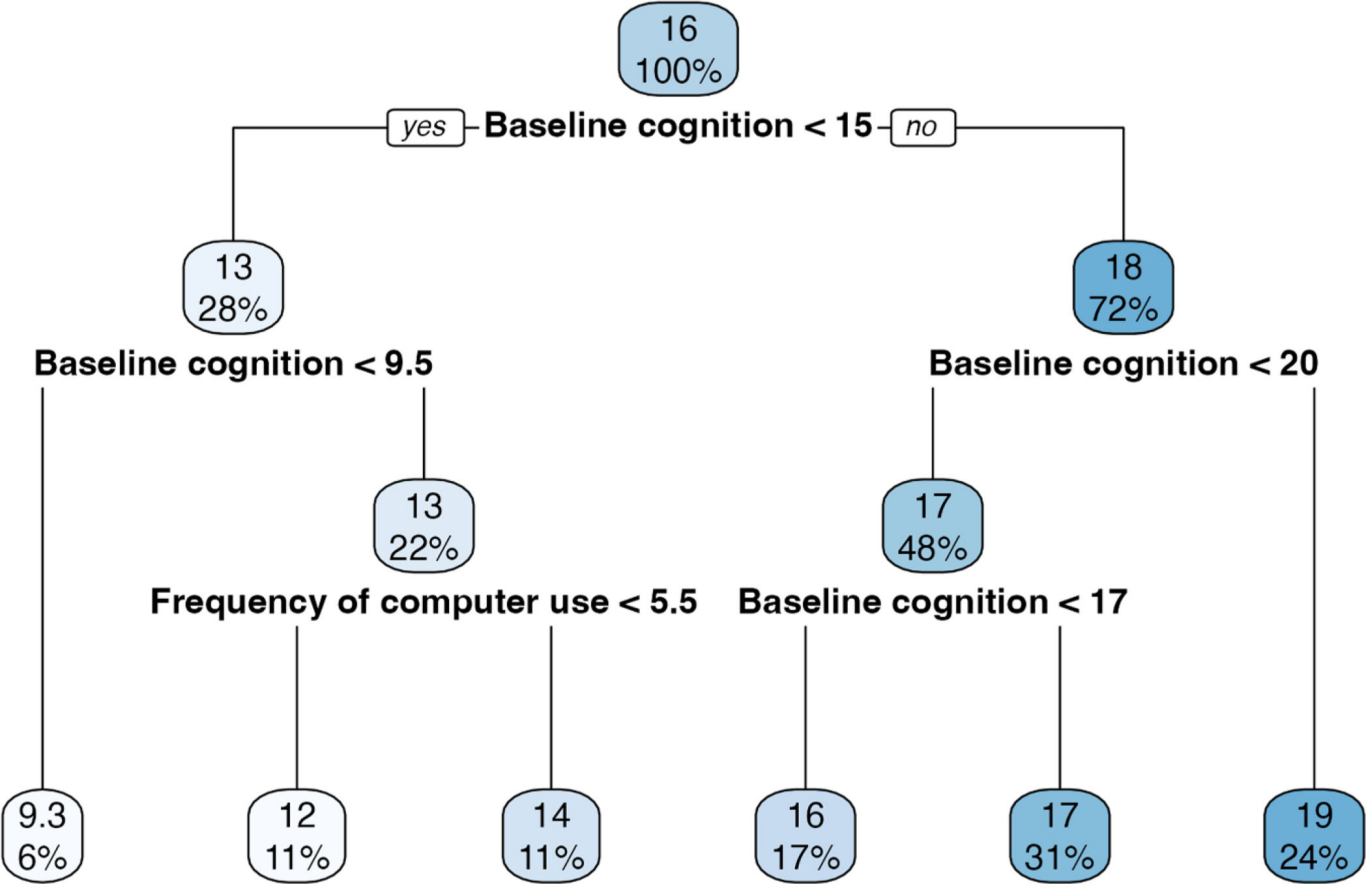
Decision tree.

The decision tree model performed the worst but identified that *baseline cognitive function score* and *frequency of computer use* explained most of the variation in follow-up cognitive function. These two variables were considered important by all other models either through significance, inclusion, or importance. However, computer use only played a small role for those with a baseline cognitive function between 10 and 14. For those in this range, their estimated follow-up cognitive function was 13, with a point subtracted if they only use the computer once a week (12) or less and a point added if they use it more (14). All other decisions were based on baseline cognitive score. If someone had <9.5 points at baseline, their predicted follow-up score would be 9.3 points; if someone had between 15 and 16 points at baseline, their predicted follow-up score would be 16 points; if someone had between 17 and 19 points at baseline, their predicted follow-up score would be 17 points; and lastly, if someone had 20 or more points at baseline, their predicted follow-up score would be 19 points. Fig. 4 illustrates the model’s decisions. The top number at each node represents the estimated follow-up cognitive function score before making the decision, while the percentage below indicates the proportion of data at that split. Surprisingly, this model does not contain age.

**Fig. 5.**
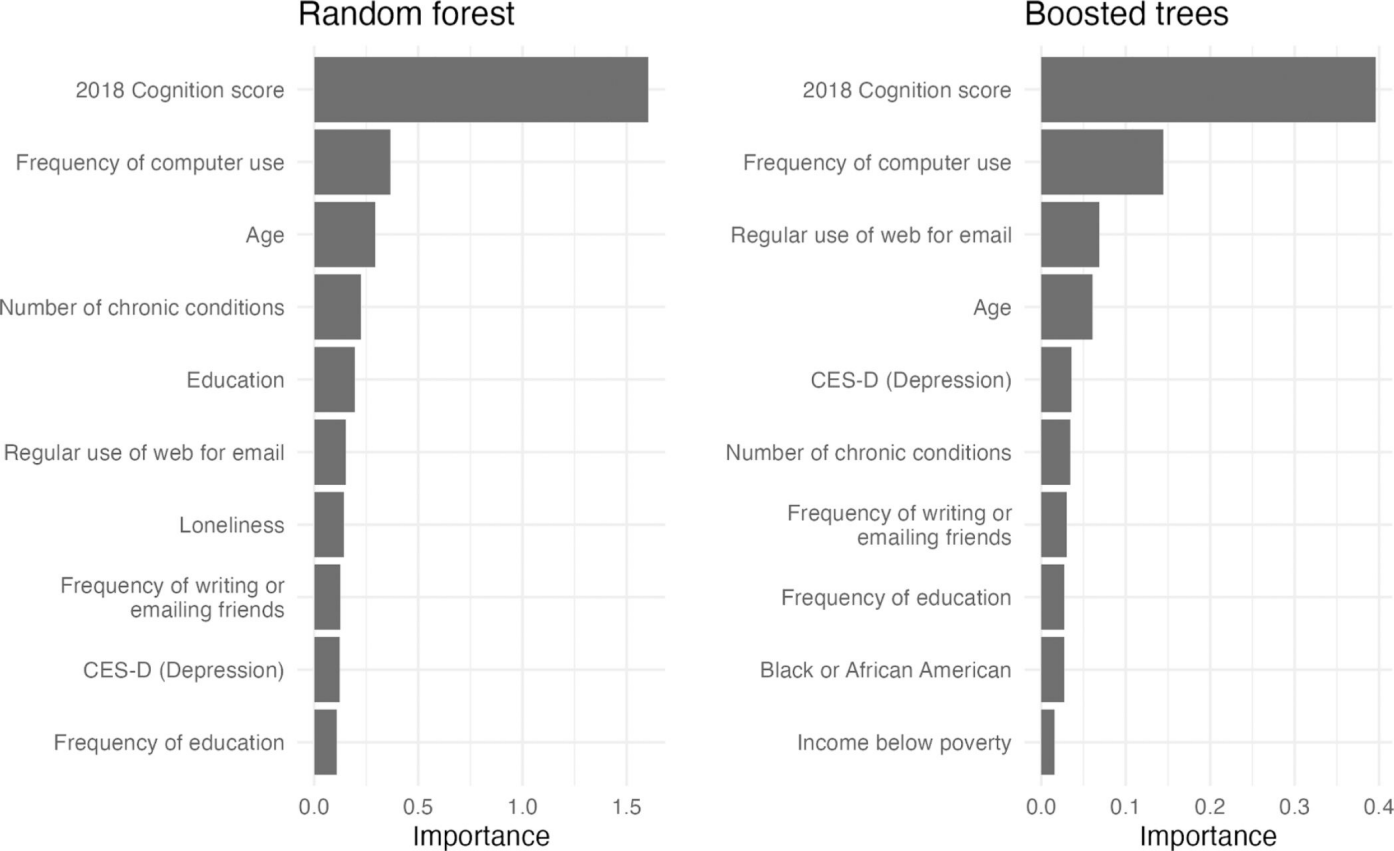
Variable importance plots of the random forest and boosted trees models using the permutation method.

Variable importance plots for the random forest and boosted trees models’ top ten important variables are shown in Fig. 5. Characteristics that appear in both models are baseline cognitive function, frequency of computer use, age, number of chronic conditions, regular use of email, depression (CES-D), and frequency of writing or emailing friends.

## Discussion

The practical goal of social science-based research is to explain some, but not all, of the variation in outcome. Therefore, Ozili (2023) proposed that regression models with R-squared values of at least 0.1 are reasonable if accompanied by significantly associated variables. Although this study’s aim was to have good predictive performance, the significant variables and R-squared values ranging from 0.402 to 0.435 are more than decent. It is clear among all models that follow-up cognitive function is largely determined by baseline condition and that lower frequency of web use is a risk factor. There were no significant differences between the best performing models, so the interpretability of the traditional regression models had more clinical importance than the tree-based models.

It is common that decision tree and MARS do not perform as well as linear regression due to their simplicity. This study’s results are comparable to a study using ML techniques to predict mortality during an acute myocardial infraction hospitalization – the logistic regression model performed the best, followed by the MARS and decision tree models. Another study using a regression model and two kinds of decision trees to predict anxiety and depression among youth found that the decision trees did not perform as well as the regression models but selected the same important variables (Battista et al., 2023). Nonetheless, cases do arise where a decision tree can outperform regression models. To predict readmission in a United Kingdom hospital, Demir (2014) used a decision tree that outperformed logistic regression and MARS. The success of the decision tree could be explained by a single node which classified all patients with an emergency readmission within the past 30 days as a future readmission. Out of the 166 patients in this node, 88 % of them were readmitted. This shows that decision trees perform best when they can generalize the data to a set of simple rules. Perhaps the cumulative effect of secondary predictors such as age, education, chronic conditions, and web use in this study is what gave the regression, ensemble, and boosted methods a slight edge in performance.

Surprisingly, the complex tree-based models did not outperform the regression models. This could mean that the data did not contain higher order terms (for example, age^2^ or age^3^) or interaction terms that are suitable for spline or ensemble methods to capture (Zhang et al., 2019). But not only is the data simple and suited for regression models, it also contains predictors which are highly correlated, thereby amplifying their effect on the model’s predictions. The elastic net model achieved better performance than the linear regression model by eliminating these redundant features, whereas decision trees and MARS simplified the data too much. Yıldırım (2024) applied many ML models to data with known multicollinearity issues and found that linear regression using the Liu estimator outperformed complex tree-based methods and MARS.

Although this study uses the permutation method to calculate variable importance, there are other model-specific and model-agnostic techniques that could be employed. Specific to the random forest model, variable importance could have been calculated by recording the *purity* at each node as the trees were being built. Purity is defined as how well the predictor at each node can separate the outcome into its true values. The better the predictor can split the outcome, the more important it is considered. The important variables calculated by this method were not shown, but they were similar to those obtained through the permutation method, with eight out of ten of the same predictors, albeit in slightly different order. On the other hand, model-agnostic techniques such as *Local Interpretable Model agnostic Explanation* (LIME) (Ribeiro et al., 2016) or *SHapley Additive exPlanations* (SHAP) (Lundberg & Lee, 2017) could be used to calculate each variable’s importance and their positive or negative relationship to the outcome. However, LIME is sensitive to changes in the input data and the locality of input features that the user wants to be explained (Bhattacharya, 2022, p. 97). Even within the same input dataset, explanations of different parts of the feature space could be completely different. If the model is non-linear or complex, SHAP may be a better method of explainability. The only caveat of SHAP is its time complexity of *O*(2^*n*^) where *n* is the number of predictors in the dataset (Bhattacharya, 2022, p. 140). Given that the data in this study was fit well to linear models, either of these variable importance methods could have been used to explain black box models such as random forest or boosted trees.

Future studies could use a longitudinal design since the HRS provides data in two-year increments. This would open the door to models that deal with repeated observations such as a linear mixed-effects model. Even within patient observations, fluctuations in scores can make detecting trends difficult unless many observations are gathered (Jonaitis et al., 2019). Global associations may exist, but individual variation and baseline characteristics must be considered to make accurate predictions relative to each individual. By using longitudinal data, interactions may arise between age and characteristics that affect cognitive trajectory.

## Conclusion

Elastic net regression provided the best balance of predictive performance, interpretability, and regularization in modeling follow-up cognitive function. The results suggest that the relationship between predictors and cognitive function is largely additive and linear, with limited benefit from modeling complex interactions. The performance gains of elastic net over standardized linear regression highlight the value of addressing multicollinearity by penalizing or removing highly correlated predictors. Notably, tree-based models such as random forest and boosted trees, while powerful in other machine learning applications, underperformed in this structured clinical dataset due to the absence of strong interaction effects and the presence of correlated features.

These findings have important implications for clinical practice, health policy, and future machine learning applications. Simpler, interpretable models not only achieve robust predictive performance but also enhance clinician trust and facilitate seamless integration into clinical workflows, reducing reliance on complex post hoc explainability tools. Furthermore, incorporating longitudinal designs that incorporate patient-specific variation may further strengthen predictive accuracy and relevance for delivering individualized, patient-centered care.

## Supplementary Material

1

2

Supplementary materials

Supplementary material associated with this article can be found, in the online version, at doi:10.1016/j.mlwa.2025.100694.

## Figures and Tables

**Table 1:** Participant characteristics.

	Unweighted	Weighted

**Characteristic**	*** N* = 4,889** ^1^	***N* = 92,312,651** ^1^
**2020 Cognitive function score** (1–27)	15.6 (4.6)	16.1 (4.5)
**2018 Cognitive function score** (1–27)	16.0 (4.1)	16.5 (4.2)
**Male**	1970 (40 %)	42,619,950 (46 %)
**Race**		
*White/Caucasian*	3546 (73 %)	74,301,342 (81 %)
*Black/African American*	884 (18 %)	9643,428 (10 %)
*Other*	447 (9.2 %)	8111,967 (8.8 %)
*Unknown*	12	
**Hispanic**	627 (13 %)	9448,658 (10 %)
*Unknown*	4	
**Age**	68 (10)	66 (9)
**Marital**		
*Married*	2770 (57 %)	56,954,211 (62 %)
*Separated*	956 (20 %)	16,897,849 (18 %)
*Divorced*	819 (17 %)	11,217,664 (12 %)
*Widowed*	335 (6.9 %)	7082,899 (7.7 %)
*Never married*	9 (0.2 %)	160,028 (0.2 %)
**Poverty**		
*Household income above poverty*	4344 (89 %)	83,454,171 (90 %)
*Household income below poverty*	545 (11 %)	8858,480 (9.6 %)
**Education**		
*No degree*	603 (12 %)	9260,950 (10 %)
*High school or GED*	2540 (52 %)	45,557,254 (49 %)
*Bachelor or associate degree*	1205 (25 %)	25,718,364 (28 %)
*Graduate or higher*	540 (11 %)	11,765,210 (13 %)
*Unknown*	1	
**Loneliness** (1–3)	1.54 (0.45)	1.54 (0.46)
*Unknown*	54	
**CES-D** (0–8)	1.29 (1.88)	1.25 (1.87)
**Positive family support** (1–12)	8.70 (2.61)	8.59 (2.63)
*Unknown*	369	
**Positive friend support** (1–12)	9.20 (2.23)	9.22 (2.23)
*Unknown*	546	
**Negative family support** (1–16)	6.36 (2.55)	6.43 (2.56)
*Unknown*	419	
**Negative friend support** (1–16)	5.66 (1.96)	5.70 (1.93)
*Unknown*	594	
**Live alone**	3253 (67 %)	62,137,221 (67 %)
**Children live within 10 miles**	2077 (50 %)	36,812,867 (48 %)
*Unknown*	760	
**Financial transfer to kids**	1622 (37 %)	32,860,923 (40 %)
*Unknown*	500	
**Financial transfer from kids**	301 (6.9 %)	4383,709 (5.4 %)
*Unknown*	495	
**Frequency of adult care** ^2^	2.02 (1.86)	1.96 (1.79)
*Unknown*	102	
**Frequency of activities with grandchildren** ^2^	3.08 (1.87)	3.08 (1.87)
*Unknown*	87	
**Frequency of youth volunteering** ^2^	1.71 (1.26)	1.72 (1.24)
*Unknown*	80	
**Frequency of charity work** ^2^	2.18 (1.57)	2.20 (1.55)
*Unknown*	86	
**Frequency of education** ^2^	1.64 (1.06)	1.69 (1.06)
*Unknown*	86	
**Frequency of sports/socializing/clubs** ^2^	2.33 (1.59)	2.44 (1.62)
*Unknown*	73	
**Frequency of non-religious activities** ^2^	1.73 (1.14)	1.75 (1.14)
*Unknown*	67	
**Frequency of computer use** ^2^	5.16 (2.51)	5.53 (2.32)
*Unknown*	55	
**Frequency of writing or emailing friends** ^2^	2.88 (1.83)	3.03 (1.81)
*Unknown*	625	
**Medicaid**	648 (13 %)	10,257,339 (11 %)
*Unknown*	26	
**Regular use of web for email**	3203 (66 %)	67,108,688 (73 %)
*Unknown*	7	
**Number of paid helpers**	0.0221 (0.1714)	0.0238 (0.1861)
**Number of unpaid helpers**	0.13 (0.46)	0.12 (0.43)
**Number of family members ever helped**	0.14 (0.50)	0.13 (0.46)
**Number of non-family members ever helped**	0.0229 (0.1806)	0.0256 (0.1980)
**Days got help last month**	2 (8)	2 (8)
*Unknown*	16	
**Hours got help last month**	8 (53)	8 (54)
*Unknown*	35	
**Days paid helpers helped last month**	0.3490 (3.0562)	0.3758 (3.3059)
*Unknown*	6	
**Hours paid helpers helped last month**	1.7336 (23.8808)	2.3304 (31.3357)
*Unknown*	9	
**Days unpaid helpers helped last month**	1.8 (7.5)	1.6 (7.1)
*Unknown*	13	
**Hours unpaid helpers helped last month**	7 (48)	6.0 (43.9)
*Unknown*	29	
**Days family helped last month**	1.8 (7.7)	1.7 (7.3)
*Unknown*	11	
**Hours family helped last month**	7 (48)	6.2 (43.8)
*Unknown*	27	
**Days non-family helped last month**	0.2737 (2.5858)	0.3091 (2.7828)
*Unknown*	7	
**Hours non-family helped last month**	1.5535 (24.0522)	2.1151 (31.2442)
*Unknown*	11	
**Number of chronic conditions**	2.30 (1.50)	2.11 (1.51)
**Difficulties with activities of daily living**	0.26 (0.76)	0.25 (0.75)
**Difficulties with instrumental activities of daily living**	0.17 (0.56)	0.18 (0.59)

1 Mean (SD); n (%).

2 Likert scales that were recoded to their numerical values for use in modeling. Their original values are as follows – (1) Never/not relevant, (2) Not in the last month, (3) At least once a month, (4) Several times a month, (5) Once a week, (6) Several times a week, and (7) Daily.

**Table 2:** Differences in baseline cognitive function score by demographics.

Characteristic	Weighted baseline cognitive function score^1^	p-value^2^

**Age**		<0.001
<*65 years*	16.6 (4.5)	
*65*+ *years*	15.5 (4.5)	
**Gender**		<0.001
*Male*	15.8 (4.4)	
*Female*	16.4 (4.7)	
**Race**		<0.001
*White/Caucasian*	16.5 (4.2)	
*Black/African American*	13.5 (5.1)	
*Other*	14.9 (5.0)	
**Hispanic**		<0.001
*No*	16.3 (4.5)	
*Yes*	14.4 (4.8)	
**Marital**		<0.001
*Married*	16.7 (4.2)	
*Separated*	15.5 (4.7)	
*Divorced*	14.5 (4.9)	
*Widowed*	14.9 (4.9)	
*Never married*	14.2 (5.5)	
**Poverty**		<0.001
*Household income above poverty*	16.4 (4.4)	
*Household income below poverty*	13.2 (4.7)	
**Education**		<0.001
*No degree*	12.1 (4.8)	
*High school or GED*	15.5 (4.2)	
*Bachelor or associate degree*	17.5 (4.1)	
*Graduate or higher*	18.0 (4.0)	

1 Mean (SD)

2 Design-based Kruskal-Wallis test.

**Table 3:** Candidate hyperparameters and their finalized values.

Model	Range of candidate hyperparameters	Number of candidates	Finalized values

Elastic net	• Penalty: [−10, 0] •Mixture: [0, 1]	20	• Penalty: 0.0886 • Mixture: 0.5
Random forest	• Number of predictors: [1, 46] •Number of trees: [1, 2000] •Minimum number of data points: [2, 40]	20	•Number of predictors: 15 •Number of trees: 2000 •Minimum number of data points: 18
Boosted trees	•Number of predictors: [1, 46] •Number of trees: [1, 2000] •Minimum number of data points: [2, 40] •Tree depth: [1, 15] •Learn rate: [−10, −1]	30	Number of predictors: 6 •Number of trees: 1448 •Minimum number of data points: 22 •Tree depth: 1 •Learn rate: 0.0356

**Table 4:** Linear regression and variables selected from the elastic net model.

	Linear regression			Elastic net*		
Characteristic	Beta	95 % CI	p-value	Beta	95 % CI	p-value

**2018 Cognitive score function**	0.50	0.47, 0.53	**<0.001**	0.50	0.47, 0.54	**<0.001**
**Male**						
*No*	—	—		—	—	
*Yes*	−0.57	−0.79, −0.35	**<0.001**	−0.60	−0.82, −0.39	**<0.001**
**Race**						
*White/Caucasian*	—	—		—	—	
*Black/African American*	−0.49	−0.86, −0.11	**0.010**	−0.44	−0.81, −0.07	**0.019**
*Other*	−0.09	−0.49, 0.30	0.639	−0.07	−0.46, 0.32	0.722
**Hispanic**						
*No*	—	—		—	—	
*Yes*	0.52	0.12, 0.92	**0.011**	0.56	0.16, 0.96	**0.006**
**Age**	−0.04	−0.06, −0.03	**<0.001**	−0.04	−0.05, −0.03	**<0.001**
**Married**						
*Married*	—	—		—	—	
*Separated*	−0.34	−0.62, −0.05	**0.021**	−0.36	−0.64, −0.08	**0.013**
*Divorced*	−0.16	−0.51, 0.19	0.362	−0.15	−0.50, 0.20	0.407
*Widowed*	−0.71	−1.2, −0.25	**0.002**	−0.70	−1.2, −0.24	**0.003**
*Never married*	0.99	−1.4, 3.4	0.413	0.98	−1.4, 3.4	0.417
**Poverty**						
*Household income above poverty*	—	—		—	—	
*Household income below poverty*	−0.17	−0.59, 0.25	0.432	−0.17	−0.59, 0.24	0.414
**Education**						
*No degree*	—	—		—	—	
*High school or GED*	0.44	0.03, 0.85	**0.035**	0.47	0.07, 0.87	**0.023**
*Bachelor or associate degree*	0.66	0.19, 1.1	**0.006**	0.70	0.24, 1.2	**0.003**
*Graduate or higher*	1.1	0.53, 1.6	**<0.001**	1.1	0.56, 1.6	**<0.001**
**Loneliness**	−0.03	−0.31, 0.25	0.859	−0.06	−0.33, 0.21	0.669
**CES-D (Depression)**	−0.14	−0.21, −0.07	**<0.001**	−0.14	−0.20, −0.07	**<0.001**
**Positive family support**	0.00	−0.04, 0.05	0.844			
**Positive friend support**	0.04	−0.02, 0.09	0.214	0.04	−0.02, 0.09	0.165
**Negative family support**	0.00	−0.05, 0.05	0.955			
**Negative friend support**	−0.03	−0.10, 0.03	0.280			
**Live alone**						
*No*	—	—				
*Yes*	−0.04	−0.28, 0.20	0.736			
**Children live within 10 miles**						
*No*	—	—		—	—	
*Yes*	−0.20	−0.43, 0.04	0.099	−0.20	−0.44, 0.03	0.087
*unknown*	−0.76	−1.2, −0.30	**0.001**	−0.80	−1.2, −0.35	**<0.001**
**Financial transfer to kids**						
*No*	—	—		—	—	
*Yes*	0.22	−0.01, 0.45	0.062	0.21	−0.01, 0.44	0.066
*unknown*	2.3	0.19, 4.4	**0.033**	1.4	0.85, 1.9	**<0.001**
**Financial transfer from kids**						
*No*	—	—				
*Yes*	−0.08	−0.56, 0.40	0.743			
*unknown*	−0.96	−3.0, 1.1	0.368			
**Frequency of adult care**	0.02	−0.04, 0.08	0.453			
**Frequency of activities with grandchildren**	0.05	−0.01, 0.11	0.115	0.05	−0.01, 0.11	0.109
**Frequency of youth volunteering**	−0.02	−0.12, 0.08	0.700			
**Frequency of charity work**	0.01	−0.07, 0.09	0.813			
**Frequency of education**	0.02	−0.09, 0.13	0.721			
**Frequency of sports/socializing/clubs**	−0.08	−0.15, −0.01	**0.031**	−0.08	−0.15, −0.01	**0.028**
**Frequency of non-religious activities**	0.09	−0.01, 0.20	0.066	0.10	0.01, 0.20	**0.039**
**Frequency of computer use**	0.15	0.08, 0.23	**<0.001**	0.16	0.08, 0.23	**<0.001**
**Frequency of writing or emailing friends**	0.06	−0.01, 0.13	0.106	0.05	−0.02, 0.12	0.179
**Medicaid**						
*No*	—	—		—	—	
*Yes*	−0.36	−0.74, 0.03	0.069	−0.35	−0.73, 0.03	0.073
**Regular use of web for email**						
*No*	—	—		—	—	
*Yes*	−0.06	−0.44, 0.32	0.765	−0.03	−0.41, 0.34	0.868
**Number of paid helpers**	−1.4	−3.0, 0.21	0.089	−0.85	−1.8, 0.11	0.083
**Number of unpaid helpers**	−0.26	−1.3, 0.77	0.622			
**Number of family members ever helped**	0.43	−0.49, 1.3	0.359			
**Number of non-family members ever helped**	1.1	−0.28, 2.5	0.115	0.36	−0.63, 1.3	0.480
**Days got help last month**	0.36	0.12, 0.60	**0.003**			
**Hours got help last month**	−0.01	−0.05, 0.03	0.617			
**Days paid helpers helped last month**	0.13	−0.52, 0.79	0.691			
**Hours paid helpers helped last month**	0.04	−0.01, 0.09	0.144	0.01	0.00, 0.01	**0.011**
**Days unpaid helpers helped last month**	0.08	−0.55, 0.72	0.795	−0.01	−0.03, 0.01	0.228
**Hours unpaid helpers helped last month**	0.04	−0.02, 0.09	0.191			
**Days family helped last month**	−0.45	−1.0, 0.12	0.125			
**Hours family helped last month**	−0.03	−0.08, 0.02	0.198			
**Days non-family helped last month**	−0.54	−1.1, 0.04	0.067	−0.04	−0.10, 0.02	0.216
**Hours non-family helped last month**	−0.03	−0.07, 0.02	0.279			
**Number of chronic conditions**	−0.15	−0.23, −0.07	**<0.001**	−0.15	−0.23, −0.08	**<0.001**
**Difficulties with activities of daily living**	0.03	−0.16, 0.21	0.782			
**Difficulties with instrumental activities of daily living**	−0.24	−0.52, 0.03	0.083	−0.24	−0.48, 0.01	0.063

Abbreviation: CI = Confidence Interval.

* Predictors that have been removed by the elastic net are greyed out.

## Data Availability

The data can be accessed through the Health and Retirement Study website at https://hrs.isr.umich.edu/about.
