# Supplementary figures and images for: Regularized regression outperforms trees for predicting cognitive function in the Health and Retirement Study
Source: Mach Learn Appl. Author manuscript; Available in PMC 2025 Nov 27. (PMC12652623; doi:10.1016/j.mlwa.2025.100694)

**Figure S1.** Distribution of baseline and follow-up cognitive function score.


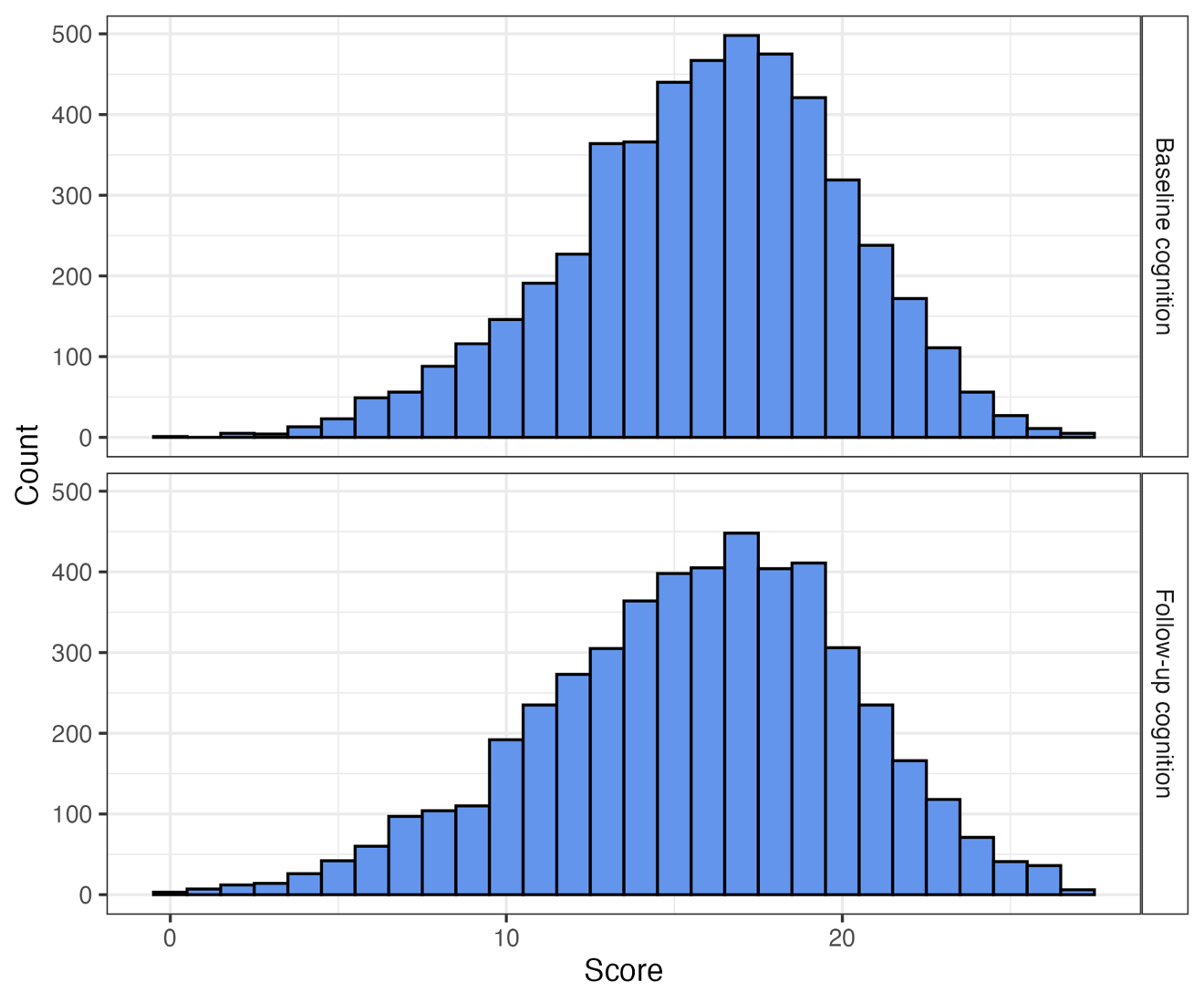

Supplement: 1 [file NIHMS2111926-supplement-1.docx]
