# Supplementary material for: Regularized regression outperforms trees for predicting cognitive function in the Health and Retirement Study
Source: Mach Learn Appl. Author manuscript; Available in PMC 2025 Nov 27. (PMC12652623; doi:10.1016/j.mlwa.2025.100694)

**Figure S2**. Regularization of the elastic net model coefficients.

*Each line represents a coefficient values of the elastic net model shrinking as the penalty value is increased. The vertical line indicates the optimal penalty parameter that minimizes the root mean squared error.*


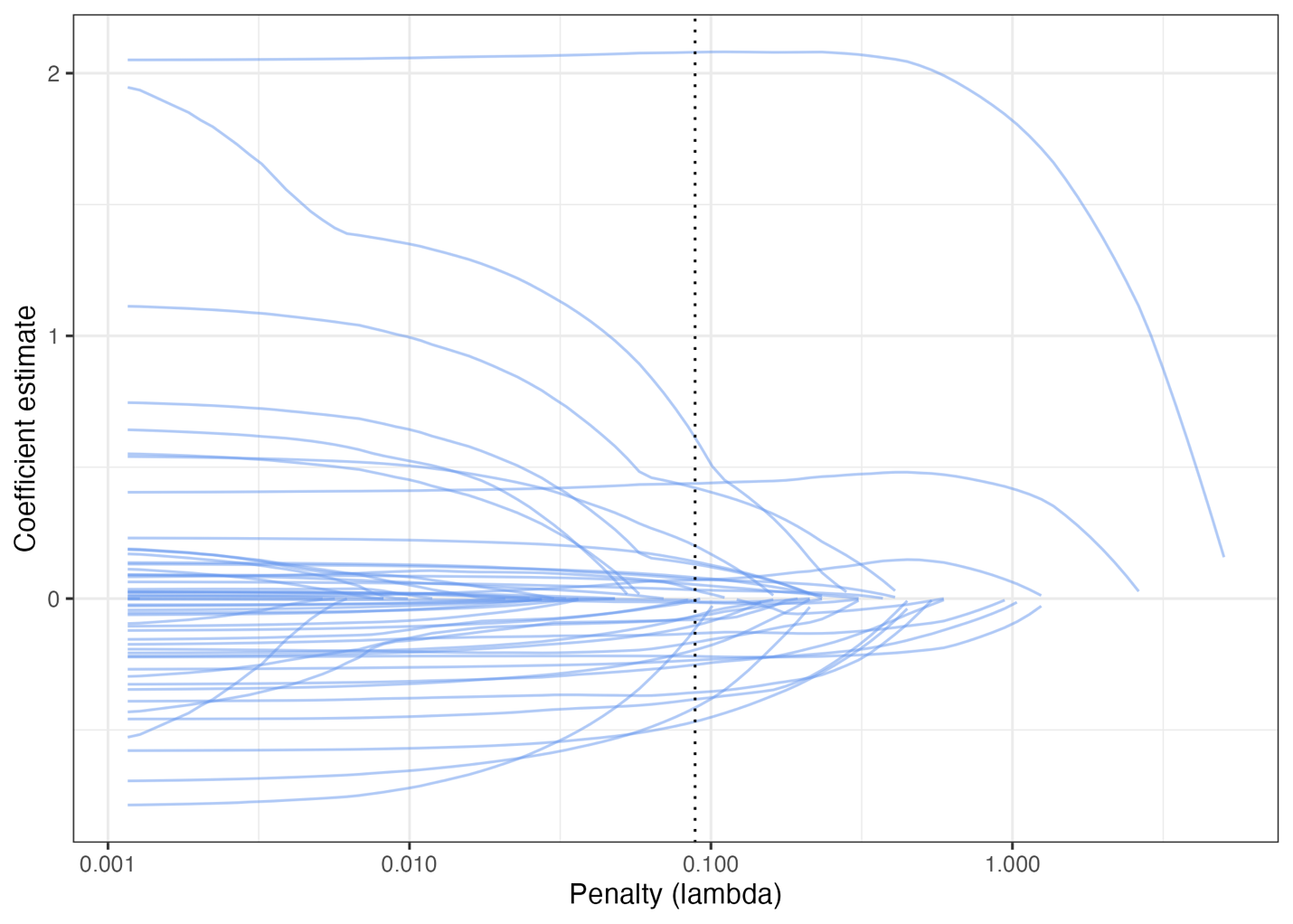

Supplement: 2 [file NIHMS2111926-supplement-2.docx]
